# Corneal Lesions Associated With Feline Herpesvirus‐1 Modified Live Vaccine Strain F2 in Two Cats

**DOI:** 10.1155/crve/8387185

**Published:** 2026-06-17

**Authors:** Yasuharu Suga, Seiya Maehara, Akihiko Saito, Yasunari Kitamura, Rikio Kirisawa

**Affiliations:** ^1^ Suga Animal Clinic, Katsushika-ku, Tokyo, Japan; ^2^ Environmental Science for Sustainable Development, Graduate School of Agricultural and Life Sciences, The University of Tokyo, Bunkyo-ku, Tokyo, Japan, u-tokyo.ac.jp; ^3^ Hikarimachi Animal Eye Clinic, Ebetsu, Hokkaido, Japan; ^4^ Triangle Animal Eye Clinic, Bunkyo-ku, Tokyo, Japan; ^5^ Yakumo Animal Hospital, Yakumo, Hokkaido, Japan

**Keywords:** corticosteroids, eosinophilic keratitis, F2 strain, feline herpesvirus-1, stromal keratitis, symblepharon

## Abstract

Feline herpesvirus‐1 (FHV‐1) modified live vaccine (MLV) is designated as a core vaccine for cats. As reported here, two 2‐month‐old domestic short hair cats (Cat 1 and Cat 2; the two were littermates) initially presented with sneezing, nasal and ocular discharge, and ocular discomfort (Day 0), which had persisted since their vaccination. At 1.5 months of age, both cats were vaccinated with a vaccine containing the FHV‐1 MLV strain F2 at another clinic. A few days later, they developed sneezing and ocular discharge almost simultaneously. Ten days after vaccination, they were diagnosed with allergic conjunctivitis and administered corticosteroid eye drops for 7 days. On Day 0, both cats presented with dendritic ulcers in one eye and a superficial corneal ulcer in the other eye. Treatment with oral antiherpesvirus medication and hyaluronic acid eye drops was initiated. A genomic analysis of FHV‐1 strains isolated on Day 0 and of specimens obtained on Day 3 for real‐time PCR suggested that both cats were infected with the same strain as the F2 strain and that there was no mixed infection with the field strain. Real‐time PCR was performed to identify ocular surface pathogens other than FHV‐1, and feline calicivirus (FCV) was detected in specimens collected from the superficial corneal ulcers of both cats on Day 3. On Day 6, dendritic ulcers in both cats healed. On Day 15, superficial corneal ulcers in both cats developed corneal crystalline deposits and deep neovascularization. In the eyes with superficial corneal ulcers in both cats, symblepharon subsequently developed in Cat 1, and eosinophilic keratitis subsequently developed in Cat 2. In conclusion, findings suggested that corneal ulcers caused by the F2 strain may be associated with the development of specific corneal lesions in cats that were young, that had a coinfection with FCV, and that were administered corticosteroids.

## 1. Introduction

Feline herpesvirus‐1 (FHV‐1) is widespread globally and is a major pathogen causing infectious ocular surface diseases (IOSD) in cats [1]. Like FHV‐1, pathogens that cause IOSD include feline calicivirus (FCV), *Chlamydia felis*, and *Mycoplasma felis* [1]; coinfection with these pathogens may exacerbate clinical signs [2]. Among the pathogens that cause IOSD, FHV‐1 causes an infection characterized by persistence and relapse. Therefore, vaccination is recommended as a part of a strategy for controlling infections [3]. In 2020, the American Animal Hospital Association and the American Association of Feline Practitioners published the Feline Vaccination Guidelines, a vaccination program designed to control FHV‐1 infection [4]. The representative strain of the FHV‐1 modified live vaccine (MLV) is the F2 strain, which has been attenuated through serial passage [5]. However, a report indicated that the F2 strain was isolated from the dendritic ulcer eye of a cat with a history of corticosteroid treatment [6]. The administration of corticosteroids is known to exacerbate ocular disease caused by FHV‐1; in experimental infection with a field strain of FHV‐1, the administration of corticosteroids has been found to cause dendritic ulcers to progress to chronic stromal keratitis characterized by geographic ulcers, interstitial edema, and deep vascularization [7]. In contrast, chronic stromal keratitis does not develop in untreated eyes [7]. In the aforementioned experiment by Nasisse et al. [7], calcium corneal deposits and corneal sequestration also developed in eyes that were treated with corticosteroids. However, there are currently no reports that have observed the course of corneal lesions due to the F2 strain as have been induced by corticosteroid administration.

Given the presented information, the aim of this report is to describe two cases of corneal lesions in cats with a documented history of vaccination, including the F2 strain, and previous corticosteroid treatment. FHV‐1 was isolated from specimens collected from both cats during the initial visit. To determine whether the infecting FHV‐1 strains corresponded to the F2 vaccine strain, a genomic analysis was performed on the isolates. In addition, the corneal lesions observed in both cats during treatment were compared with those previously reported in cats experimentally infected with a field strain of FHV‐1 [7].

## 2. Case Presentation

### 2.1. Cat 1

Cat 1 was an approximately 2‐month‐old intact male domestic shorthair. The primary clinical signs were persistent sneezing, nasal and ocular discharge, and ocular discomfort, which started following vaccination. The owner informed that Cat 1 was approximately 1 month of age when it was picked up from the outdoors and appeared to be in good health. Fourteen days later, the cat was administered the MLV F2 strain (Purevax RCP, Nippon Boehringer Ingelheim Co. Ltd., Tokyo, Japan) at another clinic. Approximately 2–3 days later, sneezing and ocular discharge were observed. Ten days after vaccination, the cat was diagnosed with allergic conjunctivitis and administered betamethasone sodium phosphate fradiomycin sulfate eye drops (Eye, nose RINDERON‐A solution, Shionogi & Co. Ltd., Osaka, Japan) q12h in both eyes for 7 days.

**Figure 1:**
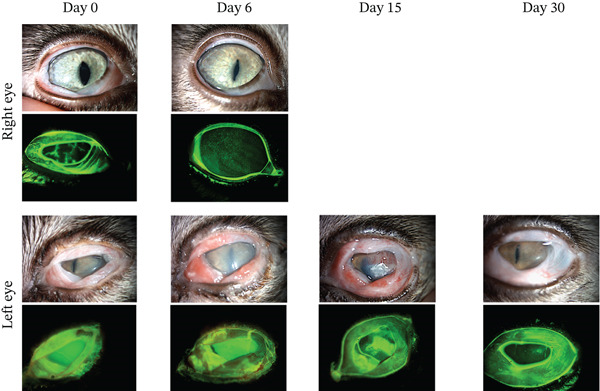
Photographs of ocular lesions in Cat 1 during the course of treatment. The lower photos are of each eye stained with fluorescein. Day 0: severe conjunctivitis in both eyes, dendritic ulcers in the right eye and an extensive superficial corneal ulcer in the left eye. Day 6: dendritic ulcers in the right eye healed. Day 15: corneal crystalline deposits and deep neovascularization, and adhesion between the bulbar or palpebral conjunctiva, cornea, and third eyelid (symblepharon) in the superficial corneal ulcer in the left eye. Day 30: The superficial corneal ulcer had healed, but the symblepharon remained.

Upon their initial visit (Day 0), Cat 1 weighed 700 g, and its body condition score (BCS) was 3/9, which is marginally below the ideal. The owner reported that Cat 1′s clinical signs worsened after the administration of corticosteroid eye drops, which had been started 7 days prior. A slit‐lamp microscopic examination (SLD‐4, Topcon Corp., Tokyo, Japan) revealed severe conjunctivitis in both eyes, dendritic ulcers in the right eye, and an extensive superficial corneal ulcer in the left eye (Figure 1). An FHV‐1 infection was suspected, so specimens were collected from both eyes and the nose for a definitive diagnosis via virus isolation prior to fluorescein staining. Sterile Dacron swabs (Texwipe, Kernersville, North Carolina, United States) were used to wipe tears from the conjunctiva of each eye to collect individual specimens. A sterile microbrush (Shofu Inc., Kyoto, Japan) was inserted into each nostril to swab the nasal mucosa and collect specimens, which were then combined into a single specimen. After specimen collection, fluorescein staining (FLUORES Ocular Examination Test Paper 0.7 mg, Ayumi Pharmaceutical Co. Ltd., Tokyo, Japan) was performed and photographs were taken (Figure 1). FHV‐1 was isolated from all of the specimens (Table 1). A strain of FHV‐1 isolated from the right eye with dendritic ulcers has been registered in GenBank (Accession No. OR514564) in a previous study [6] (Table 1). The corneal ulcers in both eyes and the rhinitis were definitively diagnosed as being caused by FHV‐1. After administering one drop of oxybuprocaine hydrochloride (Benoxil ophthalmic solution, Santen Pharmaceutical Co. Ltd., Osaka, Japan) to anesthetize the ocular surface, a sterile microbrush (Shofu) was then used to obtain a cytology sample from the superficial corneal ulcer. No bacteria were observed. Treatment with oral famciclovir (Daiichi Sankyo Espha, Co. Ltd., Tokyo, Japan) 90 mg/kg q12 was administered as antiherpesvirus therapy, and treatment with one drop of 0.3% hyaluronic acid eye drops (Santen) q8h was initiated in both eyes to promote repair of the ocular surface. The SNAP FIV/FeLV Combo Test (IDEXX) yielded negative results for feline immunodeficiency virus (FIV) and feline leukemia virus (FeLV).

**Table 1:** The specimen collection site, collection date, and results of virus isolation and genomic analysis for each cat.

Case	Sampling site (finding of the lesion)	Day 0			Day 3			
		Virus isolation	FHV‐1		Real‐time PCR for FHV‐1	FHV‐1		Real‐time PCR for pathogens causing IOSD
			ORF28	ORF44		ORF28	ORF44	
Cat 1	Right eye (dendritic ulcer)	FHV‐1^a^	+^b^	+^b^	Positive	/	/	/
	Left eye (superficial corneal ulcer)	FHV‐1	/	/	Positive	+	+	FCV
	Nose	FHV‐1	/	/	Positive	+	+	/

Cat 2	Right eye (superficial corneal ulcer)	N.D.	/	/	Positive	+	N.A.	FCV
	Left eye (dendritic ulcer)	N.D.	/	/	Positive	+	+	/
	Nose	FHV‐1	+	+	Positive	/	/	/

*Note:* The “+” in the table indicates the presence of a single‐nucleotide variant in the F2 strain marker, and the absence of nucleotide overlap.

Abbreviations: FHV‐1, feline herpesvirus‐1; IOSD, infectious ocular surface disease; N.A., not analyzable; N.D., not detected; ORF, open reading frame.

^a^This FHV‐1 strain has been registered with GenBank by Suga and Kirisawa [6] (Accession Number OR514564).

^b^A genomic analysis has been previously conducted by Suga and Kirisawa [6].

As of Day 3, the results of the virus isolation from the specimens collected on Day 0 had yet to return. The cat was a kitten, so there was a possibility that the specimen was not collected in a sufficient amount for virus isolation. Moreover, given the extensive and severe superficial corneal ulcer in the left eye, there was a possibility of coinfection with pathogens that cause IOSD other than FHV‐1. Therefore, we obtained the owner′s approval to perform real‐time PCR, which is more sensitive than pathogen isolation, to detect FHV‐1 [8] and other pathogens that cause IOSD including FCV, *C. felis*, and *M. felis*. Real‐time PCR for FHV‐1 was performed using primers targeting thymidine kinase, with specimens collected from both eyes and the nose using the same method as for virus isolation (Canine‐Lab Corp., Tokyo, Japan). All of the specimens yielded positive results (Table 1). Real‐time PCR for pathogens that cause IOSD other than FHV‐1 was performed on a specimen collected from the superficial corneal ulcer eye (Canine‐Lab): FCV was detected (Table 1). On Day 6, dendritic ulcers in the right eye healed (Figure 1), and clinical signs of rhinitis also improved. On Day 15, corneal crystalline deposits and deep neovascularization were observed in the superficial corneal ulcer in the left eye (Figure 1). These findings were similar to those observed in eyes that were administered corticosteroids during experimental infection with the field strain [7]. On the same day, an adhesion between the bulbar or palpebral conjunctiva, cornea and third eyelid (symblepharon) was also observed (Figure 1). On Day 30, the superficial corneal ulcer had healed, but the symblepharon remained (Figure 1).

### 2.2. Cat 2

Cat 2 was a littermate of Cat 1 and an intact male, and lived with Cat 1. As in Cat 1, the primary clinical signs in Cat 2 were persistent sneezing, nasal and ocular discharge, and ocular discomfort, which started following vaccination. The vaccination history, clinical course, and history of corticosteroid treatment of Cat 2 prior to Day 0 were similar to those of Cat 1.

**Figure 2:**
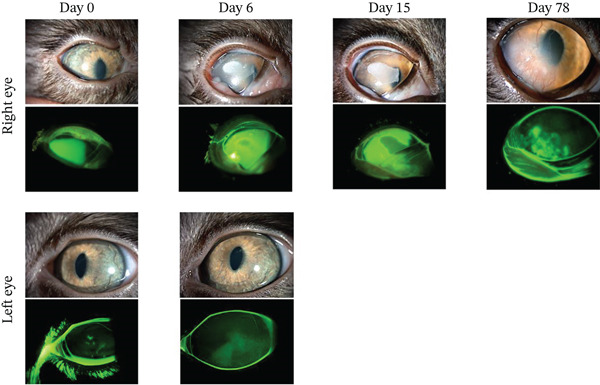
Photographs of ocular lesions in Cat 2 during the course of treatment. The lower photos are of each eye stained with fluorescein. Day 0: mild conjunctivitis in both eyes, an extensive superficial corneal ulcer in the right eye, and dendritic ulcers in the left eye. Day 6: dendritic ulcers in the left eye healed; corneal crystalline deposits in the superficial corneal ulcer in the right eye. Day 15: deep neovascularization in the superficial corneal ulcer. Day 78: infiltrative lesion found at the temporal corneal limbus of the eye with the superficial corneal ulcer.

On Day 0, Cat 2 weighed 720 g and its BCS was 3/9, which is marginally below the ideal. The owner reported that Cat 2′s clinical signs worsened after the administration of corticosteroid eye drops, similar to Cat 1. Examinations similar to those performed on Cat 1 revealed mild conjunctivitis in both eyes, an extensive superficial corneal ulcer in the right eye, and dendritic ulcers in the left eye (Figure 2). As with Cat 1, swabs of both eyes and the nose were collected for virus isolation. FHV‐1 was not isolated from the specimens collected from both eyes, but it was isolated from a specimen collected from the nose (Table 1). The rhinitis was definitively diagnosed as being caused by FHV‐1. As with Cat 1, a cytology of the superficial corneal ulcer was performed, but no bacteria were observed. The same treatment as administered to Cat 1 was initiated. The SNAP FIV/FeLV Combo Test (IDEXX) yielded negative results for both viruses.

On Day 3, real‐time PCR for FHV‐1 was performed on specimens collected from both eyes and the nose (Canine‐Lab), as with Cat 1. All of the specimens yielded positive results (Table 1). Taking into consideration the diagnosis of Cat 1, the corneal ulcers in both eyes of Cat 2 were definitively diagnosed as being caused by FHV‐1. Real‐time PCR for pathogens that cause IOSD other than FHV‐1 was performed on a specimen collected from the superficial corneal ulcer eye (Canine‐Lab): FCV was detected (Table 1). On Day 6, dendritic ulcers in the left eye healed (Figure 2), and clinical signs of rhinitis also improved. The superficial corneal ulcer in the right eye was observed to have corneal crystalline deposits on Day 6 and deep neovascularization on Day 15 (Figure 2). These findings were similar to those in Cat 1. They were also similar to those observed in eyes that were administered corticosteroids during experimental infection with the field strain [7]. The treatment continued and the superficial corneal ulcer decreased in size over time, but it did not heal. On Day 78, an infiltrative lesion was observed at the temporal corneal limbus of the eye with the superficial corneal ulcer (Figure 2). Cytology of the lesion revealed eosinophils, leading to a diagnosis of eosinophilic keratitis.

### 2.3. Genomic Analysis

In a previous study, next‐generation sequencing revealed that the FHV‐1 strain (OR514564) isolated from Cat 1′s eye with dendritic ulcers was identical to the F2 strain, and a genomic analysis revealed no evidence of mixed infection with the field strain (Table 1) [6]. A genomic analysis was also performed on the isolate from the nose of Cat 2 on Day 0. In that analysis, single‐nucleotide variants (SNVs) in open reading frame (ORF) 28 and ORF44 of the F2 strain (1280A and 397G, respectively) were used as markers for simplified genotyping of the F2 strain [6]. PCR was performed on the isolate from the nose of Cat 2, with primers specific to ORF28 and ORF44, and it was then sequenced [6]. A total of two SNVs that are markers for the F2 strain were found, one in ORF28 and one in ORF44, in the isolate (Table 1). No nucleotide overlap was observed at these positions (data not shown) (Table 1).

With the owner′s approval, a genomic analysis was performed on the remaining specimens after real‐time PCR on Day 3. That analysis used the same methods as were used to analyze the isolate from the nose of Cat 2. Specimens collected from the eye with a superficial corneal ulcer and the nose of Cat 1 and from the eye with dendritic ulcers and the eye with a superficial corneal ulcer of Cat 2, which had not been included in the genomic analysis of isolates, were subjected to a genomic analysis. A total of two SNVs that are markers for the F2 strain were found in all of the specimens except for the eye with a superficial corneal ulcer of Cat 2 (Table 1). No nucleotide overlap was observed at these positions (data not shown) (Table 1). Although the specimen from the eye with a superficial corneal ulcer of Cat 2 had the SNV in ORF28, and no nucleotide overlap was observed at that position, the amount of DNA in ORF44 that was amplified with PCR was not sufficient for sequencing (Table 1).

## 3. Discussion

The F2 strain was isolated from two cats with corneal ulcers, corneal crystalline deposits, and deep neovascularization (Cat 1 and Cat 2), symblepharon (Cat 1), and eosinophilic keratitis (Cat 2) were observed during their course of treatment. In addition to both cats being administered corticosteroids, they were also young and coinfected with FCV. The findings of this report suggested that corneal ulcers caused by the F2 strain may be associated with the development of specific corneal lesions in cats that had risk factors that could potentially exacerbate clinical signs of FHV‐1 infection.

In the current genomic analysis, there was no evidence of mixed infection with the field strain in any other specimens, with the exception of one instance where the ORF44 could not be analyzed using the specimen collected from Cat 2′s eye with a superficial corneal ulcer. Therefore, the results of the previous [6] and current genomic analyses suggest that both cats were infected with the same strain as the F2 strain and that there was no mixed infection with the field strain. However, the specimen that was collected from Cat 2′s eye with a superficial corneal ulcer on Day 3 yielded a minimal amount of DNA in ORF44 that was amplified with PCR, so analysis of the SNV of the F2 strain marker in ORF44 was not possible. Moreover, FHV‐1 was not isolated from either of Cat 2′s eyes on Day 0. For some unknown reason, the conjunctivitis in Cat 2′s eyes was milder than that in Cat 1′s eyes. FHV‐1 preferentially replicates in the conjunctiva rather than the cornea [7], so the speculation is that there may have been only a small amount of FHV‐1 in the specimens collected from Cat 2′s eyes, which could have precluded virus isolation on Day 0 and a genetic analysis on Day 3.

The corneal crystalline deposits and deep neovascularization observed during the course of treatment of the superficial corneal ulcer in both cats in this report were similar to the findings in eyes following the administration of corticosteroids (subconjunctival injection of betamethasone) during experimental infection with the field strain [7]. According to that study, corneal crystalline deposits (calcium corneal deposits) may result from a secondary degenerative response related to the severe corneal stromal damage caused by corticosteroid administration and corneal infection with the field strain. Since both cats in this report were infected with the F2 strain and had been administered corticosteroid eye drops containing betamethasone sodium phosphate for 7 days, the corneal crystalline deposits in both cats may likewise have been associated with a severe corneal ulcer caused by corticosteroid administration and corneal infection with the F2 strain. Although corneal neovascularization is a common, nonspecific response to injury or inflammation of the cornea [9], the exact pathogenesis of corneal crystalline deposits in cats is unclear, and the condition is rare [10]. In humans, frequent administration of hyaluronic acid containing a high concentration of phosphate (at least six times a day) has been reported to lead to corneal calcification [11]. Unlike during experimental infection with the field strain [7], both cats in this report received 0.3% hyaluronic acid eye drops (one drop three times a day) to promote the healing of corneal ulcers. However, the hyaluronic acid eye drops manufactured by Santen Pharmaceutical that were used in this report do not contain phosphate, so this treatment is unlikely to have caused the corneal crystalline deposits. A study has been reported that calcium corneal deposits in cats may be associated with systemic comorbidities, including chronic kidney disease, calcium oxalate lithiasis, and suspected hypercalcemia, in addition to ocular surface conditions such as corneal ulcers, dryness, and topical treatment [10]. Both cats in this report were slightly underweight (BCS of 3/9) on Day 0. However, they exhibited a robust appetite and steadily gained weight throughout the course of treatment. Moreover, they showed no clinical signs of suspected systemic comorbidities. Consequently, although a blood biochemistry and urinalysis were not performed, systemic comorbidities were surmised to not be a contributing factor to the development of the corneal crystalline deposits.

A symblepharon is a condition in which the conjunctiva adheres to itself and/or the cornea as a result of a severe FHV‐1 ocular infection [12]. On Day 0, Cat 1 presented with an extensive superficial corneal ulcer and severe conjunctivitis, both of which likely contributed to the adhesion of these lesions. Therefore, the symblepharon observed in Cat 1 may be associated with that resulting from conjunctivitis and a corneal ulcer caused by the F2 strain. In corneal scrapings from cats with eosinophilic keratitis, FHV‐1 was detected in 76% of specimens via PCR, suggesting that FHV‐1 may be associated with the pathogenesis of eosinophilic keratitis [13]. Moreover, up to 24% of cats diagnosed with eosinophilic keratitis also had corneal ulcers [14]. In Cat 2, the superficial corneal ulcer caused by the F2 strain was observed to have not healed when eosinophilic keratitis was diagnosed, suggesting a possible association between the corneal ulcer caused by the F2 strain and the development of eosinophilic keratitis. That said, both cats in this report similarly developed corneal crystalline deposits and deep neovascularization during the course of treatment of a superficial corneal ulcer, but Cat 1 subsequently developed symblepharon instead of eosinophilic keratitis. The development of eosinophilic keratitis may be associated with a type I or type IV hypersensitivity reaction [15]. Therefore, the speculation was that a hypersensitivity reaction may have been associated with the development of eosinophilic keratitis in Cat 2, whereas a hypersensitivity reaction did not occur in Cat 1. However, there are no data indicating differences in both cats′ immune responses, so the reason why they developed different ocular lesions remains unknown.

Corneal sequestration developed in five of 10 eyes treated with corticosteroids during the course of experimental infection with the field strain [7], but it did not develop in either cat in this report. The exact cause of corneal sequestration remains unknown, but it has been reported to develop more frequently after grid keratotomy to treat nonhealing corneal ulcers [16]. In experimental infection with the field strain, severe epithelial injury was induced by using a 25‐gauge needle to facilitate corneal infection during FHV‐1 instillation [7], a method equivalent to grid keratotomy. However, neither of the cats in this report had undergone grid keratotomy, so corneal sequestration may not have developed. Therefore, whether or not grid keratotomy has been performed may be implicated in the development of corneal sequestration, and whether the difference in the virulence between the F2 strain and the field strain contributed to the development of this disease is unclear.

Similar to the cats experimentally infected with the field strain [7], both cats in this report were administered corticosteroids. However, both cats were approximately 2 months of age on Day 0, which was younger than the cats experimentally infected with the field strain (6–8 months of age) [7]. The clinical signs caused by an FHV‐1 infection may be exacerbated in young cats [12], so evaluation of the virulence of the F2 strain was not possible in this report. Moreover, FCV that was detected via real‐time PCR in both cats on Day 3 may have exacerbated ocular lesions due to a coinfection with FHV‐1 [2]. However, FCV was not isolated from either cat in any of the virus isolations performed on Day 0. Isolating pure FHV‐1 via virus isolation using samples collected from cats infected with both FHV‐1 and FCV is generally difficult, as FCV proliferates relatively faster [17]. Therefore, the FCV detected via real‐time PCR on Day 3 in both cats may be a remnant of a previous infection. Both cats in this report lived outdoors until they were approximately 1 month of age, and there were no other cats in their household environment after they were picked up. Therefore, the FCV infection is likely to have occurred outdoors. If the infection occurred outdoors immediately before the cats were picked up, it may have been associated with the clinical signs that appeared 2–3 days after vaccination. However, these clinical signs may have been caused by the reactivation of a latent F2 strain following vaccination [6]. That said, many cats continue to shed the virus after recovering from FCV infection, and in most of them this viral shedding may persist for at least 30 days [18]. Therefore, both cats in this report may have already recovered from the FCV infection and were thus asymptomatic when they were picked up, but they were still shedding the virus. However, the period of FCV shedding was coming to an end, so the speculation is that there was no infectious FCV present in the virus isolation on Day 0, and the FCV detected via real‐time PCR on Day 3 was considered to be a viral remnant. Nevertheless, the lack of viral testing prior to Day 0, which would have determined the stage at which FCV was involved in the ocular lesions, represents a limitation of this report. That said, as seen in both cats in this report, young cats that are coinfected with FHV‐1 and FCV are often encountered clinically [17]. A point worth noting is that administering corticosteroids to such cats, even if they are infected with the F2 strain rather than the field strain, may lead to corneal lesions and also be associated with refractory ocular conditions such as symblepharon and eosinophilic keratitis.

In conclusion, this report suggests that corneal ulcers caused by the F2 strain may be associated with the development of corneal crystalline deposits, deep neovascularization, symblepharon, and eosinophilic keratitis in cats that were young, that had a coinfection with FCV, and that were administered corticosteroids.

## Funding

No funding was received for this manuscript.

## Disclosure

This paper was presented in part at the 2023 Japanese Society of Clinical Veterinary Medicine Symposium.

## Consent

Written consent has not been obtained from the owner, as there are no patient identifiable data included in this case report. However, verbal consent was obtained from the owner to use the specimens for a genomic analysis.

## Conflicts of Interest

The authors declare no conflicts of interest.

## Data Availability

All data relevant to the cases are included in this article.
